# The nomogram for the prediction of overall survival in patients with metastatic lung adenocarcinoma undergoing primary site surgery: A retrospective population-based study

**DOI:** 10.3389/fonc.2022.916498

**Published:** 2022-08-15

**Authors:** Chao Ma, Shuzhen Peng, Boya Zhu, Siying Li, Xiaodong Tan, Yaohua Gu

**Affiliations:** ^1^ School of Public Health, Wuhan University, Wuhan, China; ^2^ Department of Health Management, Huang pi District People’ Hospital, Wuhan, China

**Keywords:** metastatic lung adenocarcinoma, overall survival, nomograms, SEER database, prognosis

## Abstract

**Background:**

Lung adenocarcinoma (LUAD) is the most common type of Non-small-cell lung cancer (NSCLC). Distant metastasis of lung adenocarcinoma reduces the survival rate. we aim to develop a nomogram in order to predict the survival of patients with metastatic lung adenocarcinoma.

**Methods:**

We retrospectively collected patients who were initially diagnosed as metastatic LUAD from 2010 to 2015 from SEER database. Based on the multivariate and univariate Cox regression analysis of the training cohorts, independent prognostic factors were assessed. The nomogram prediction model was then constructed based on these prognostic factors to predict the overall survival at 12, 24 and 36 months after surgery. Nomogram were identified and calibrated by c-index, time-dependent receiver operating characteristic curve (time-dependent AUC) and calibration curve. Decision curve analysis (DCA) was used to quantify the net benefit of the nomogram at different threshold probabilities, and to better compare with the TNM staging system, we calculated the c-index of this nomogram as well as the net reclassification improvement (NRI) and the integrated discrimination improvement (IDI).

**Result:**

A total of 1102 patients with metastatic LUAD who met the requirements were included for analysis. They were randomly divided into 774 in the training cohorts and 328 in the validation cohorts. As can be seen from the calibration plots, the predicted nomogram and the actual observations in both of the training and validation cohorts were generally consistent. The time dependent AUC values of 12 months, 24 months and 36 months were 0.707, 0.674 and 0.686 in the training cohorts and 0.690, 0.680 and 0.688 in the verification cohorts, respectively. C-indexes for the training and validation cohorts were 0.653 (95%CI 0.626-0.68)and 0.663 (95%CI 0.626-1), respectively. NRI and IDI show that the model is more clinical applicable than the existing staging system. In addition, our risk scoring system based on Kaplan Meier (K-M) survival curve can accurately divide patients into three hierarchy risk groups.

**Conclusion:**

This has led to the development and validation of a prognostic nomogram to assist clinicians in determining the prognosis of patients with metastatic lung adenocarcinoma after primary site surgery.

## Introduction

As one of the most common forms of cancer, lung cancer has a high mortality rate among the 13 regions of the world indicating that lung cancer is a serious threat to human health and life (1). According to recent research, non-small cell lung cancer (NSCLC) accounts for about 85% of all lung cancers among all its subtypes. The prevalence of lung adenocarcinoma (LUAD), the most common subtype of NSCLC, is still on the rise among current, former, and even non-smokers (2).

It is estimated that the 5-year relative survival rate of patients with LUAD is only 5% due to the fact that about 57% of the patients have advanced stage and metastatic disease (3). A lung cancer patient’s prognosis is adversely affected by the presence of distant metastases (4). Lung, bone, brain, adrenal gland, pleura, and liver are the general metastatic sites for LUAD (5). Since the turn of the century, there have been tremendous advances in the treatment of non-small cell lung cancers (NSCLC), with the overall survival rate of patients using immunotherapy and targeted biologic regimens far exceeding that of those treated with conventional cytotoxic chemotherapy in the past (6). While there have been significant developments in surgical techniques and adjuvant therapies in recent years, the overall prognosis of LUAD remains very poor despite the recent advances.

A tumor-node-metastases (TNM) staging system is one of the main indicators used in predicting survival and determining treatment options (7). Although TNM stage is similar in patients with different survival rates. Furthermore, there are other patient-specific factors that are associated with survival in multiple cancers, including age, race, marital status, tumor size, and differentiation (8). The development of an improved staging prediction system that considers the characteristics of the tumor with the patient’s own condition is therefore essential.

In clinical practice, nomograms are widely used in terms of prognosis for cancer as a simple statistical tool (9). By evaluating the weighted prognostic value for each of the factors, the probability of an event can be calculated (10). As an alternative or even as a new standard, nomograms have been proposed for many cancers that compare favorably with the traditional TNM staging system (11).

Consequently, the objective of this study is to identify prognostic factors that are relevant to LUAD after surgery by examining data in the Surveillance, Epidemiology, and End Results (SEER) database of LUAD patients and to develop a prediction model for survival after surgery. An evaluation of the prediction model was assessed using calibration curves, receiver operating characteristic (ROC) curves, and decision curve analyses (DCA) (12).

## Materials and methods

### Study population and data source

The data for the patients were obtained through the US SEER database using SEER*Stat software(version 8.3.6; National Cancer Institute, USA).Participants should meet the following criteria to be considered for inclusion in the study:(1) diagnosed with pulmonary adenocarcinoma by histology from 2010 to 2015; (2) patients with distant metastasis at the initial diagnosis; (3)received primary site surgery (4) patients with important variable information being fully documented. This study excluded patients diagnosed by autopsy or death certificate. In addition, patients with survival time < 1 month were also excluded. A random sample of patients selected for the study was divided into two groups, a training group (70%) and a testing group (30%).

A human subject or any personally identifiable information was not included in the data. Consequently, informed consent was not required for this portion of the study.

### Study variables

In this study, 15 variables were included to identify independent prognostic factors in patients with postoperative patients with metastatic LUAD. The demographic variables include age, race, gender and marital status. The clinicopathological characteristics of the tumor include primary location, laterality, tumor grade(I,II,III,IV), N stage, T stage,liver metastasis, brain metastasis, lung metastasis, bone metastasis and treatment information, including chemotherapy and radiotherapy. Among the primary outcomes of this study was overall survival (OS). It was defined as the interval between the date of diagnosis and the date of death from whatever cause. Using X-tile software, we determined the best age and survival cut-off values (**Supplementary Figure 1A**).

For age, 54 and 67 years were determined to be the best cut-off values. We categorized the patients into three groups to facilitate data processing (< 54 years old, 54-67 years old, > 67 years old). Additionally, the T stage can be divided into the following stages: T0, T1, T2, T3, and T4. It was discussed that the N stage consisted of N0 (No), N1-N3 (Yes). The M stage was defined as M0 for no metastasis and M1 for positive metastasis. We used the eighth edition of the TNM staging system to define T, N, and M for patients in the clinical stage.

### Statistical analysis

The R software randomly divided all patients into training and verification cohorts according to a ratio of 7:3. For the purpose of comparing the variables between the training cohort and the validation cohort, we used Chi-square test. In order to determine the independent prognostic factors of postoperative patients with metastatic LUAD, we first used univariate Cox regression analysis, and the variables with P < 0.05 were included in subsequent multivariate Cox regression analysis. The variables with P < 0.05 were identified as independent prognostic factors. The nomogram prediction model was then constructed based on these prognostic factors to predict the overall survival at 12-, 24- and 36-months after surgery. We explored the differences in survival rates between patients with different subtypes of lung adenocarcinoma based on the analysis of KM survival curves of the invasive subtypes included in the study.

To compare the predicted event and the actual event, calibration plots were drawn for the 12-, 24-, and 36-month OS probability. In order to assess the model’s ability to distinguish between events and nonevents, receiver operating characteristic curves (ROC) and area under curves (AUC) were used. In addition, ROC curves or time-varying ROC curves of all independent variables were generated and compared with the AUC values of the corresponding nomogram. We established a calibration curve with a decision curve analysis (DCA) curve for evaluating the nomogram. A comparison of the nomogram with the conventional TNM staging system was based on calculating the C-index. In addition, we also calculated the net reclassification improvement (NRI) and the integrated discrimination improvement (IDI) of the nomogram. Finally, according to the best cut-off value of risk score, patients in the training cohorts and verification cohorts were divided into high-risk group, medium risk group and low-risk group, and the logarithmic rank test of Kaplan Meier (K-M) survival curve was performed to verify the prognostic value of nomogram. According to the calculated total points of each patient from the nomogram, a risk classification system was created by using X-tile program for Postoperative patients with metastatic lung adenocarcinoma. All statistical analysis was conducted using SPSS 25.0 and R software (version 3.6.1) software. P <0.05 (bilaterally) was regarded as statistically significant in this study.

## Results

### Patient characteristics

In this study, 1102 postoperative patients with metastatic LUAD were included based on the inclusion and exclusion criteria The overall patient screening and study process is shown in **Supplementary Figure 2**. The training cohorts consisted of 774 patients while the validation cohorts comprised 328 patients. See **Table 1** for details. As can be seen from the Chi square test, the deviation between the training cohorts and verification cohorts was completely random, and the variable difference between the two cohorts wasn’t statistically significant.

**Table 1:** Demographics and clinical characteristics of the participants.

Characteristics	Training cohorts N (%)	Validation cohorts N (%)	Total N (%)	P-Value
**Age(year)**				0.893
<54	130 (16.80)	47 (14.33)	177 (16.06)	
54-67	354 (45.73)	157 (47.87)	511 (46.37)	
>67	290 (37.47)	124 (37.80)	414 (37.57)	
**Gender**				0.218
Male	338 (43.67)	162 (49.39)	500 (45.37)	
Female	436 (56.33)	166 (50.61)	602 (54.63)	
**Chemotherapy**				0.980
Yes	493 (63.70)	211 (64.33)	704 (63.88)	
No/unknown	281 (36.30)	117 (35.67)	398 (36.12)	
**Grade**				0.997
I	98 (12.65)	42 (12.80)	140 (12.70)	
II	318 (41.09)	135 (41.16)	453 (41.11)	
III	352 (45.48)	147 (44.82)	499 (45.28)	
IV	6 (0.78)	4 (1.22)	10 (0.91)	
**Laterality**				0.879
right	447 (57.75)	184 (56.10)	631 (57.26)	
left	327 (42.25)	144 (43.90)	471 (42.74)	
**Metastasis site**				0.794
Liver metastasis	37 (4.35)	11 (3.10)	48 (3.97)	
Lung metastasis	215 (25.23)	85 (23.94)	300 (24.86)	
Bone metastasis	122 (14.32)	52 (14.65)	174 (14.42)	
Brain metastasis	221 (25.94)	91 (25.63)	312 (25.85)	
Uncommon^1^	257 (30.16)	116 (32.68)	373 (30.90)	
**Marital**				0.617
Unmarried	310 (40.05)	121 (36.89)	431 (39.11)	
married	464 (59.95)	207 (63.11)	671 (60.89)	
**N stage**				0.967
N0	406 (52.46)	168 (51.22)	574 (52.09)	
N1	94 (12.14)	44 (13.41)	138 (12.52)	
N2	229 (29.59)	92 (28.05)	321 (29.13)	
N3	45 (5.81)	24 (7.32)	69 (6.26)	
**Race**				0.648
Black	89 (11.50)	29 (8.84)	118 (10.71)	
White	603 (77.91)	259 (78.96)	862 (78.22)	
Other	82 (10.59)	40 (12.20)	112 (11.07)	
**Radiotherapy**				0.517
No/unknown	477 (61.63)	190 (57.93)	667 (60.53)	
Yes	297 (38.37)	138(42.07)	435 (39.47)	
**Primary location**				0.956
Upper lobe, lung	439 (56.71)	183 (55.79)	622 (56.45)	
Middle lobe, lung	40 (5.17)	19 (5.79)	59 (5.35)	
Lower lobe, lung	216 (27.91)	99 (30.18)	315 (28.58)	
Other	79 (10.21)	27 (8.24)	106 (9.62)	
**T stage**				0.979
T1	125 (16.15)	60 (18.29)	185 (16.79)	
T2	225 (29.07)	97 (29.57)	322 (29.22)	
T3	192 (24.81)	81 (24.70)	273 (24.77)	
T4	232 (29.97)	90 (27.44)	322 (29.22)	

^1^ Sites of distant metastases are uncommon.

### Histological subgroup analysis of lung adenocarcinoma

We have updated the histological typing of lung adenocarcinoma in the seer database according to the histological subtype classification system for LUAD proposed by the International Association for the Study of Lung Cancer, the American Thoracic Society and the European Respiratory Society in 2011 (13). The reason for this replacement is that the seer database uses the histological typing published in 2004, which is no longer in use. The study included 1102 patients, but patients with unspecified histologic staging were excluded, and 186 patients who had subtypes or variants of invasive adenocarcinoma were selected, and survival curves were plotted for most type (**Table 2**). According to a survival analysis conducted for each subgroup type of lung adenocarcinoma, the papillary and solid subtypes showed significantly poor survival rates when compared to the lepidic and acinar subtypes of cancer. Among the included pathological subtypes, the colloid-predominant subtype exhibited the worst survival (**Supplementary Figure 3**).

**Table 2:** Correlation of the Predominant Histologic Subtype or Variant.

Predominant Histologic Subtype/Variant	N	(%)
Lepidic-predominant	31	16.67
Acinar-predominant	44	23.65
Papillary-Predominant	39	20.97
Colloid predominant	58	31.18
Solid predominant with mucin production	14	7.53

### Nomogram variables screening

In order to identify independent prognostic factors in LUAD patients, we performed univariate and multivariate Cox proportional hazards regression analysis. On the basis of a Univariate Cox regression analysis, the prognostic factors of patients with metastatic LUAD included age, gender, race, N stage, primary location, differentiation grade, tumor size, bone metastasis, liver metastasis, radiotherapy, and chemotherapy. In a multivariate Cox regression analysis, we found age, gender, primary location, N stage, bone metastasis, liver metastasis, radiotherapy, and chemotherapy significantly correlated with the postoperative prognosis of postoperative LUAD patients **(** **Table 3** **)**.

**Table 3:** Univariate and Multivariate analysis of variables for OS in patients.

Characteristics	Univariate analysis		Multivariate analysis	
	HR (95%CI)	P-value	HR (95%CI)	P-value
**Age(year)**				
<54	Reference	–	Reference	–
54-67	1.54(1.16-2.04)	0.003	1.55(1.16-2.08)	0.003
>67	2.21(1.66-2.94)	<0.001	2.47(1.84-3.31)	<0.001
**Gender**				
Male	1.25(1.05-1.50)	0.013	1.28(1.06-1.54)	0.0085
Female	Reference	–	Reference	–
**Chemotherapy**				
Yes	0.79 (0.66-0.95)	0.011	0.67(0.55-0.82)	<0.001
No/unknown	–	–	–	–
**Grade**				
I	Reference	–	Reference	–
II	1.03(0.77-1.38)	0.836	1.00(0.74-1.34)	0.9849
III	1.35(1.02-1.79)	0.037	1.29(0.96-1.73)	0.0958
IV	0.90(0.28-2.89)	0.866	0.71(0.22-2.30)	0.5707
**Laterality**				
right	0.91(0.76-1.09)	0.291	–	–
left	Reference	–	Reference	–
**Liver metastasis**				
Yes	1.75(1.21-2.53)	0.003	1.77(1.21-2.59)	0.0033
**Lung metastasis**				
Yes	0.96(0.79-1.17)	0.678	–	–
**Bone metastasis**				
Yes	1.54(1.23-1.93)	<0.001	1.35(1.07-1.71)	0.0111
**Brain metastasis**				
Yes	1.06(0.87-1.30)	0.541	–	–
**Marital**				
Unmarried	1.12 (0.93-1.34)	0.226	–	–
married	reference		reference	
**N stage**				
N0	reference		reference	
N1	1.42(1.07-1.87)	0.014	1.71(1.28-2.29)	<0.001
N2	1.55(1.27-1.90)	<0.001	1.71(1.38-2.12)	<0.001
N3	2.51(1.78-3.55)	<0.001	2.44(1.69-3.54)	<0.001
**Race**				
Black	reference		reference	
White	1.02(0.77-1.34)	0.904	1.07(0.81-1.42)	0.6391
Other	0.67(0.45-0.99)	0.045	0.74(0.50-1.11)	0.1449
**Radiation**				
No/unknown	reference		reference	
Yes	1.20(1.01-1.44)	0.043	1.40(1.14-1.71)	0.001
**Site**				
Upper lobe, lung	reference		reference	
Middle lobe, lung	0.78(0.50-1.22)	0.276	0.86(0.54-1.35)	0.508
Lower lobe, lung	1.14(0.93-1.40)	0.200	1.24(1.01-1.53)	0.0413
Other	1.45(1.09-1.93)	0.010	1.42(1.06-1.91)	0.02
**T stage**				
T1	reference		reference	
T2	1.03(0.78-1.37)	0.827	–	–
T3	1.29(0.97-1.71)	0.083	–	–
T4	1.11(0.84-1.47)	0.471	–	–

### Nomogram construction and validation

**Figure 1:**
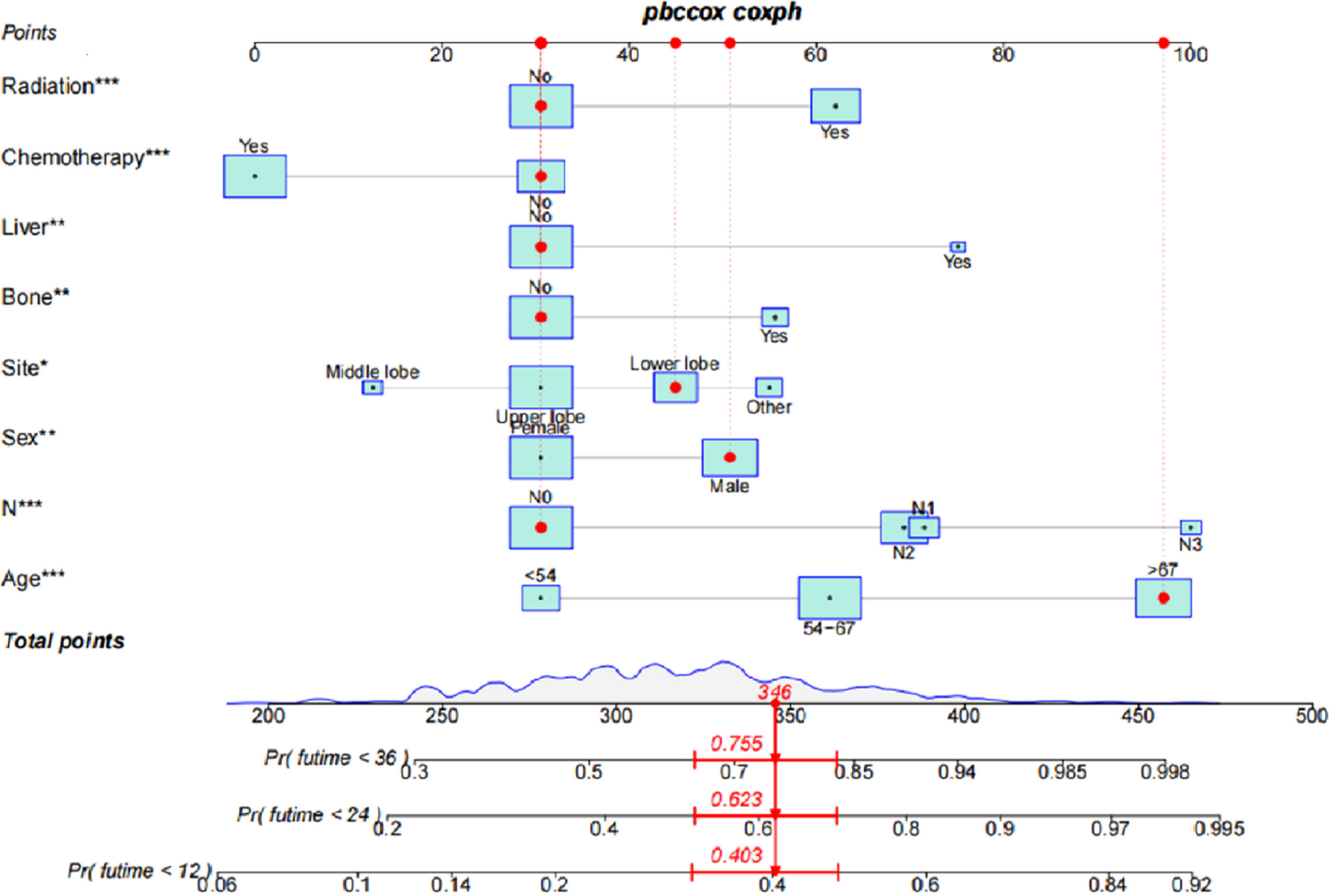
Based on standard deviations along nomogram scales, each variable was ranked according to its importance. On each variable axis, a specific point (black dot) represents the individual patient. The red lines and points indicate the number of points received by each variable. The total number of these points (346) appears on the Total Points(TP) axis, and a line appears on the survival axes to indicate the probability of 12-months survival 59.7% (1-40.3%), 24-months 37.7% (1-62.3%) and 36-months 24.5% (1-75.5%) overall survival OS:1-pr (fu time<*). * Means that 12months, 24months and 36months can be selected in OS calculation.

We constructed a nomogram for postoperative patients with metastatic LUAD according to the selected independent prognostic factors. In order to construct the model, we used these eight variables. Following this, a nomogram was constructed based on the training cohorts for predicting 12-, 24-, and 36-month OS (**Figure 1**). A point is assigned to each variable on the nomogram, and the total point can be determined by summing up the scores. In **Figure 1**, the red lines illustrate how the nomogram may be used to estimate the chance of survival of a given patient. The patient’s total risk score was established using the individual scores calculated using the nomogram; the majority of patients in this study had a total risk score ranging between 200 and 400.

**Figure 2:**
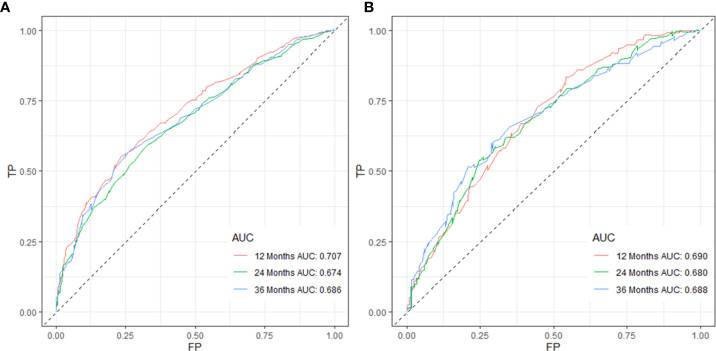
Validation of the nomogram model using 12-, 24-, and 36-month ROC curves in training **(A)** and validation cohorts **(B)**.

**Figure 3:**
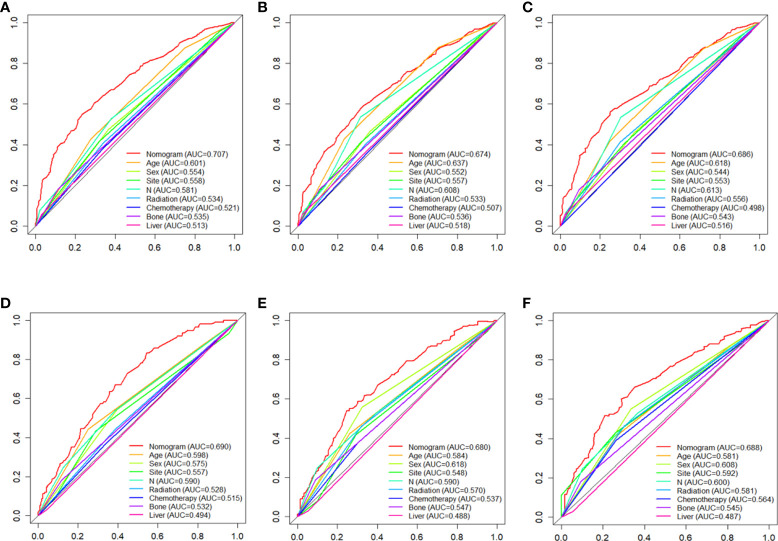
the AUC values of nomograms in the train cohorts **(A–C)** and validation cohorts **(D–F)** at 12, 24 and 36 months were compared with the AUC values of all independent factors.

**Figure 4:**
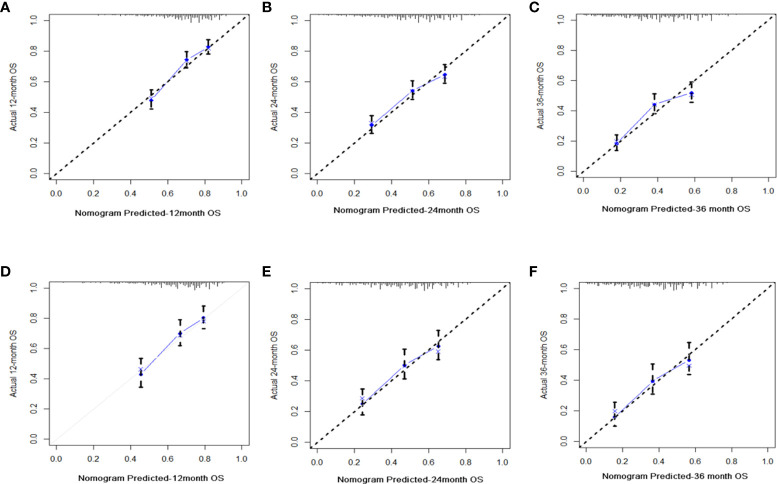
Diagrams of calibration plots of OS-associated nomograms from training and validation cohorts. Figures showing calibration plots for 12-, 24-, and 36-month OS in the training cohorts **(A–C)** and calibration plots for 12-, 24-, and 36-month OS in the validation cohorts **(D–F)**.

**Figure 5:**
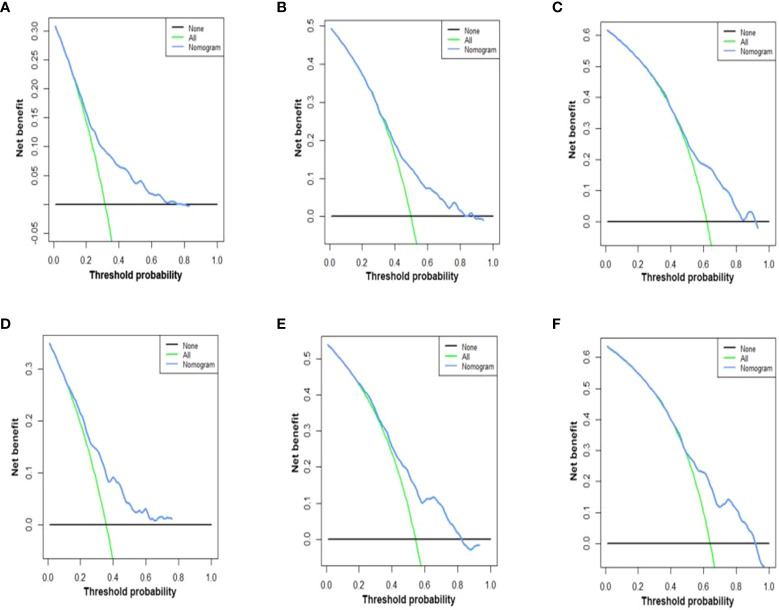
DCA of the nomograms for OS in both training and validation cohorts. The DCA of nomogram in training cohorts for both OS; **(A–C)** the DCA of nomogram in validation cohorts for OS **(D–F)**. DCA, decision curve analysis; OS, overall survival. The y-axis represents the net benefit; the x-axis represents the threshold probability. The blue line represents the net advantage of the column line graph. The black line represents the hypothesis that all patients die at 12 months, 24 months, and 36 months; the green line represents the hypothesis that no patients die at 12 months, 24 months, and 36 months.

C-index values in the training cohorts was 0.653 (95%CI 0.626-0.68) and in the validation cohorts they was 0.663 (95%CI 0.626-1).The time dependent AUC values of 12 months, 24 months and 36 months were 0.707, 0.674 and 0.686 in the training cohorts and 0.690, 0.680 and 0.688 in the verification cohorts, respectively. (**Figure 2**) Our next step was to compare nomograms and independent prognostic factors. According to our findings, the AUC of the nomogram was significantly higher than that of all independent factors at 12 months, 24 months, and 36 months, both for training and verification cohorts (**Figure 3**). A good correlation was observed between the predicted and observed survival probabilities around both the training and validation cohorts as determined by the calibrating curves of the nomogram (**Figure 4**). As a result of DCA, it was demonstrated that the new nomogram is superior to both the all-treatment and all-no-treatment regimens in predicting survival in patients with lung cancer. (**Figure 5**) Based on DCA curves, nomograms were able to better predict OS at 12, 24, and 36 months from training and validation cohorts, and at most threshold ranges, nomograms remained beneficial over both treating all patients and none treating patients at all.

### Clinical value of the nomogram compared with the TNM staging

Compared to the FIGO criteria-based tumor staging system, the nomogram was found to offer clinical benefits for predicting tumor behavior. The discrimination ability of the nomogram and the eighth version of the TNM staging system was compared in the training and validation cohorts using the IDI and NRI indices (see in **Table 4**) Compared with the eighth edition of the TNM staging system, the discriminatory power of the nomogram is significantly improved. (IDI for the 24-,36-and 48-month OS were 0.272 (p < 0.001),0.164((p < 0.001) and 0.220 (p < 0.001), respectively) and reclassification ability (continuous NRI for 24-,36-and 48-month OS were 0.272,0.164 and 0.220 respectively (both p < 0.001)) in the training group (**Table 4**).

**Table 4:** NRI and IDI of the prognostic nomogram for LUAD compared with TNM staging system.

Index	Training set			Validation set		
	Estimate	95%CI	p-value	Estimate	95%CI	p-value
**NRI (vs. AJCC TNM staging)**						
For 24-month OS	0.272	0.144- 0.427	<0.001	0.281	0.134-0.440	<0.001
For 36-month OS	0.164	0.045-0.318	<0.001	0.204	0.006-0.391	<0.001
For 48-month OS	0.220	0.093- 0.347	<0.001	0.260	0.011-0.475	<0.001
**IDI (vs. AJCC TNM staging)**						
For 24-month OS	0.084	0.071-0.097	<0.001	0.112	0.087-0.137	<0.001
For 36-month OS	0.085	0.071-0.099	<0.001	0.110	0.086-0.134	<0.001
For 48-month OS	0.083	0.070-0.096	<0.001	0.105	0.082-0.128	<0.001

### The prognostic nomogram in the clinical practice

**Figure 6:**
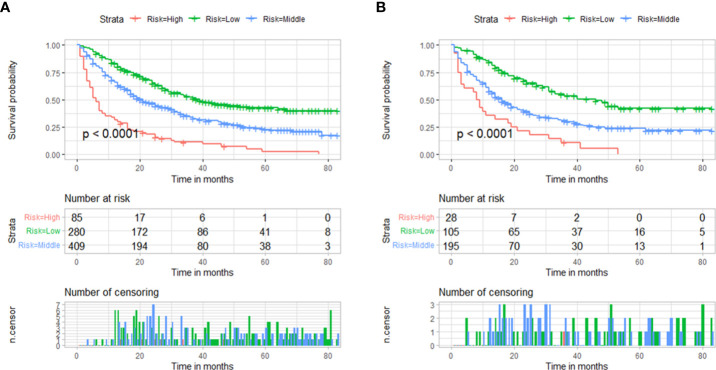
Based on the total points of the nomogram, survival curves are categorized according to risk scores (low-risk <303; middle-risk 303-372; high-risk ≥372). **(A)** Training cohorts’ survival curves. **(B)** validation cohorts’ survival curves.

Using the nomogram we were able to calculate a risk stratification based on the total points. Based on the X-tile analysis, 303 and 372 scores were selected as the optimal cutoff points (**Supplementary Figure 1B**). Three groups of patients were identified based on their risk scores: low risk (total points < 303), middle risk (303≤ total points < 372), and high risk (total points ≥ 372). Based on Kaplan-Meier OS curves, there is a great deal of discrimination between the three risk groups. If the patient is classified into the low-risk subgroup, there is always a chance that their prognosis will be better. It is evident that the prognosis of patients in the high-risk group is worse than the prognosis of patients in the low-risk group, indicating that the risk classification system based on the nomogram is an effective predictor of patients’ survival after surgery for metastatic lung cancer. A significant difference (P<0.001) was evident in **Figure 6** when comparing the survival curves in both training and validation cohorts.

### Development of a web server for accessing the new model

We have developed an online version of this nomogram **(** **Supplementary Figure 4** **)** at https://shubei11.shinyapps.io/webnomogram2/that can assist clinicians in reducing the risk of interventions and predicting survival for patients with metastatic LUAD.

## Discussion

LUAD is the most common type of NSCLC and tends to show a poor prognosis after metastasis has occurred. In early-stage patients, standard surgery is usually used to treat localized or early-stage disease, but in advanced cases, conventional therapies are usually used, such as chemotherapy and radiation, to treat the disease, and mortality rates are generally high (14).

In the last few years, the discovery of oncogenic driver mutations and their role in predicting response to targeted therapy has changed the way clinicians treat patients with LUAD (6). EGFR is the most prevalent targeted mutation in lung adenocarcinoma, and four FDA-approved tyrosine kinase inhibitors (TKIs) are currently in use. One of the drugs in first-line therapy is oxitinib, which reduces the risk of progression or death by 54% compared to earlier TKIs (erlotinib, gefitinib), and in the metastatic setting, EGFR inhibitor therapy improves PFS and quality of life for patients compared to chemotherapy (15–18). Although targeted therapies have shown promising results, almost all patients -eventually experience continued disease progression due to acquired resistance, and the induction of cell death and thus acquisition of broad resistance against targeted therapies has become a focus of research.

There have been several immunotherapy regimens used to treat patients with metastatic non-small cell lung cancer (NSCLC), but over the past decade, they have mainly focused on anti-programmed death protein-1 (PD-1) and anti-programmed death ligand 1 (PD-L1) therapies, as well as immune checkpoint blockade therapy (ICB) used to treat these patients (19). The treatment of metastatic NSCLC with two anti-PD-1 molecules and one anti-PD-L1 has been approved by the FDA (20). A new adjuvant therapy, immune checkpoint inhibitors (ICI), which can be administered alone or in combination with chemotherapy, has been developed, along with ongoing research into the level of immunotherapy, which has been shown to significantly reduce systemic recurrences and may improve long-term survival in patients with resectable NSCLC (21).

Individualized treatment is clearly becoming a paradigm for the treatment of patients with metastatic lung adenocarcinoma. To choose the right treatment, you’ve got to analyze histological features, individual genetic characteristics (mutations), antitumor drug resistance, tumor microenvironment, etc. With this personalized information, treatment will be greatly facilitated, and the nomogram we have created is one of the tools to provide information on patient survival. Hence, it is of paramount importance to study the prognosis of postoperative patients with metastatic LUAD.

In a survival analysis of different pathological subgroups of lung adenocarcinoma patients, it was found to be more likely that those patients who had predominantly lepidic and acinar forms of the disease would survive longer than those with predominantly solid and papillary forms. Other previous studies have confirmed these findings, with colloid-predominant or solid-predominant subtypes of lung adenocarcinoma often representing a poorer prognosis, suggesting that different subtypes of lung cancer can also predict survival in any given patient to some extent (22, 23). PD-L1 expression was significantly higher in papillary and solid types of LUAD than in lepidic and acinar types, according to a study assessing its expression in LUAD. A statistically significant correlation has been found between expression of PD-L1 and shorter disease-free survival outcomes and lymph node metastasis in LUAD (24). In one aspect, this also reflects the different degree of PD-L1 expression in different subtypes of lung adenocarcinoma patients, which may lead to the emergence of different survival profiles. This is the direction of research that we need to focus on in the future.

There are limitations to the current TNM staging systems in predicting mortality in cancer patients. As a result, it is essential to identify patients at high risk level after surgical resection (7). The nomogram is a necessary aspect of modern medical decision-making. The use of a nomogram carefully constructed to address a particular question, when interpreted and applied appropriately, can prove extremely useful for patients and clinicians (25). This study represents the first study that has developed and validated a nomogram model for predicting overall survival in a group of patients with metastatic lung adenocarcinoma following surgery. According to the study of 1102 patients with metastatic LUAD treated by surgery from the SEER database, such factors as age, gender, primary site, N stage, bone metastasis, liver metastasis, radiotherapy, and chemotherapy predicted postoperative survival for patients with metastatic LUAD. A survival prediction model was developed using variables that were all relatively easy to identify clinicopathologically. By including these factors in the model, the variability of patient data collection is minimized and the model’s clinical usefulness is greatly increased. The use of an easy-to-use scoring system across a wide range of settings is more likely to improve the performance of clinical assessments (26). By incorporating these independent risk factors, we developed a nomogram that accurately predicts OS in patients with metastatic LUAD at 12, 24 and 36 months. On both the training and validation cohorts, the nomogram model performed well in terms of discrimination and prediction accuracy. Multiple factors, including demographic and clinicopathological characteristics, are integrated into the nomogram to form a quantitative model which has excelled over conventional staging systems, such as the American Joint Committee on Cancer (AJCC) staging system in predicting prognosis and making clinical decisions (27).

Accordingly, the best cutoff value for OS was determined using the X-tile software, in order to divide the patients into three subgroups (low risk, middle risk, and high risk). Results from the Kaplan-Meier and Cox hazard ratio models showed significant differences between the three groups. It has been proven that nomogram can identify patients in the high-risk group more accurately than other staging systems. High-risk groups suffer from poor prognoses compared to patients at lower risk levels. In fact, it is vital to pay more attention to patients with total points≥372 than to those without.

All the patients we included have undergone surgery at the primary site, and there is no similar prognostic study for these patients. NAKAZAWA et al. results suggested that existing liver and bone metastasis adversely affected the outcome of the disease (28). The presence of liver metastases was found to be the worst prognostic factor in patients with metastatic lung adenocarcinoma (29). As stated by RIIHIMÄKI et al, patients with liver and bone metastases had a decreased survival rate when they had metastatic lung cancer (30). Based on the results of a retrospective study (31) of LUAD patients with multiple metastases, AD patients without liver metastases (4 months vs 3 months; OS and LCSS, p < 0.001) and SCLC patients (6 months vs 4 months; OS, p = 0.017; LCSS, p = 0.023) had better outcomes than patients with liver metastases. Our study included bone metastasis as an independent prognostic factor and liver metastasis as an independent prognostic factor. The overall survival of patients with LUAD with liver metastasis were worse than patients with bone metastasis.

Traditional staging of TNM has always included high N as having a poor prognosis, which is also reflected in our prognostic model. The results of a study on 167 patients with non-small cell lung cancer who underwent complete resection showed a 5-year survival rate of 20% for patients classified as N1 and 21% for patients classified as N2, with no significant differences in survival between the two groups of patients (32). It is noteworthy that our model indicated that patients with stage N1 had a better prognosis than patients with stage N2, but the difference between the two was not significant. There might be a difference in this phenomenon due to the sample population included in the study, but it is also possible to attribute it to the pre-operative and post-operative treatment the patients were given and the clinician’s surgical technique. The primary location of the tumor was also included for the first time as an independent prognostic factor in our analyses, which had not been done previously. Patients with cancer of unknown primary site (CUP) are considered to have a poor prognosis compared to patients with metastatic cancers with clear primary sites (27). Our results indicate that patients with other unknown primary sites also have poor outcomes.

Taking these results into account, we suggest that baseline characteristics in patients with metastatic LUAD, such as gender and age, can be considered independent prognostic factors. In previous retrospective studies (33, 34), we observed clearly different outcomes for men and women with NSCLC, in terms of presentation, management, and outcome. The gender of patients has been confirmed to be an independent unfavorable prognostic factor for survival in NSCLC patients. One of the most significant prognostic factors affecting lung cancer survival is the age of the patient, and the mortality rate was higher in elderly patients (35). We also got similar results about the influence of two independent prognostic factors of gender and age on OS in postoperative patients with LUAD. Men and patients older than 67 years old have a poor prognosis. There is a good probability that older patients are more susceptible to chronic diseases and postoperative complications which may severely affect their chances of survival. Second, older patients generally have poorer health, which can make surgeons hesitate to treat them as aggressively and intensively as younger patients, resulting in undertreatment of older patients (36).

All patients in this study underwent surgical treatment at the primary site, so the operation was not included in the nomogram, which does not mean that the surgical treatment has no effect on the survival rate of patients. For stage IV NSCLC patients with one or more synchronous metastases, a surgical strategy combined with systemic therapy, such as radiotherapy or chemotherapy can be effective (5, 37).

Adjuvant chemotherapy and adjuvant radiotherapy are controversial factors. Patients with NSCLC are usually treated with chemotherapy that uses platinum-based drugs. Radiation therapy, if necessary, is often used in conjunction with chemotherapy. The need to improve the long-term survival of patients with locally advanced NSCLC indicates the importance of a combination therapy, in which chemotherapy is a crucial component of controlling distant metastases in order to increase their overall survival rates (38). Cancer patients who have had stage II or stage III NSCLC completely resected survive longer when treated with adjuvant chemotherapy (39). This study also reached a similar conclusion that postoperative patients receiving adjuvant chemotherapy have a better prognosis (40–42).

An analysis of a previous meta-analysis reported that postoperative chest radiotherapy has an overall negative effect on survival, resulting in a 21.3% relative increase in odds of dying [hazard ratio (HR)1.21 (95% CI 1.08-1.34)]; meaning, an absolute reduction of 7% in survival from 55% to 48% at 2 years owing to the decrease in survival rates possible (43, 44). One of the possible reasons for this finding may be the increase in intrinsic and acquired radioresistance among patients suffering from cancers, which has led to a decrease in the success of their treatment (45).LUAD is not particularly sensitive to radiation, and thus, tumor cells are capable of developing a tolerance to radiation, resulting in local recurrences and poor prognoses (46).

There is enough sample size in SEER database to collect large sample data for research, which makes the results of this study very convincing. Even so, there will always be a certain amount of limitations associated with this study. In the first place, since this is a retrospective study, there will always be some effect of bias. Second, we cannot make a quantitative study on the specific chemotherapy and radiotherapy schemes adopted by patients and the relevant data of surgical margins state. Third, the seer database lacks information on specific tumor markers that could improve prognostic accuracy, as well as certain hematological indicators: neutrophils, platelets, and absolute lymphocyte values. Finally, there is a lack of clinical external validation data to evaluate our nomogram. Despite these limitations, our first online version of the nomogram and nomogram for surgically resected metastatic LUAD patients has high clinical applicability and provides individualized and accurate survival predictions for each patient.

## Conclusions

The survival rate of patients with metastatic LUAD after primary site surgery was predicted using a nomogram. Compared with other staging systems, the model has good prediction accuracy and clinical utility, and can provide a reference for clinicians to formulate treatment plans.

## Data availability statement

Publicly available datasets were analyzed in this study. This data can be found here: https://seer.cancer.gov/.

## Author contributions

XT, CM, and SP contributed to conception and design of the study. BZ organized the database. CM performed the statistical analysis. CM wrote the first draft of the manuscript. YG, BZ, SL, and SP wrote sections of the manuscript. All authors contributed to manuscript revision, read, and approved the submitted version.

## Conflict of interest

The authors declare that the research was conducted in the absence of any commercial or financial relationships that could be construed as a potential conflict of interest.

## Publisher’s note

All claims expressed in this article are solely those of the authors and do not necessarily represent those of their affiliated organizations, or those of the publisher, the editors and the reviewers. Any product that may be evaluated in this article, or claim that may be made by its manufacturer, is not guaranteed or endorsed by the publisher.

## Supplementary material

Supplementary Figure 1 X-tile program settings and results.

Supplementary Figure 2 The flowchart of patient selection.

Supplementary Figure 3 Survival curve analysis of major histological subtypes and variants of invasive lung adenocarcinoma. Histology (1:Acinar-predominant;2:Colloid predominant;3:Lepidic-predominant;4:Papillary-Predominant;5:Solid predominant with mucin production)

Supplementary Figure 4 Online web server interface for dynamic prognostic nomograms for patients with metastatic LUAD.

## Abbreviations

LUAD, Lung adenocarcinoma; NSCLC, Non-small-cell lung cancer; DCA, Decision curve analysis; ROC, Receiver operating characteristic; DCA, Decision curve analysis; TNM, Tumor-node-metastases; OS, Overall survival; PORT, Postoperative radiation therapy; HR, Hazard Ratio; CI, Confidence Interval; AJCC, American Joint Committee on Cancer; AUC, area under curves; K-M, Kaplan Meier; TKIs, tyrosine kinase inhibitors; PD-1, anti-programmed death protein-1; PD-L1, anti-programmed death ligand 1; ICB, immune checkpoint blockade; ICI, immune checkpoint inhibitors.
