# Supplementary figures and images for: The nomogram for the prediction of overall survival in patients with metastatic lung adenocarcinoma undergoing primary site surgery: A retrospective population-based study
Source: Front Oncol. 2022 Aug 15;12:916498. doi: 10.3389/fonc.2022.916498 (PMC9413074; doi:10.3389/fonc.2022.916498)

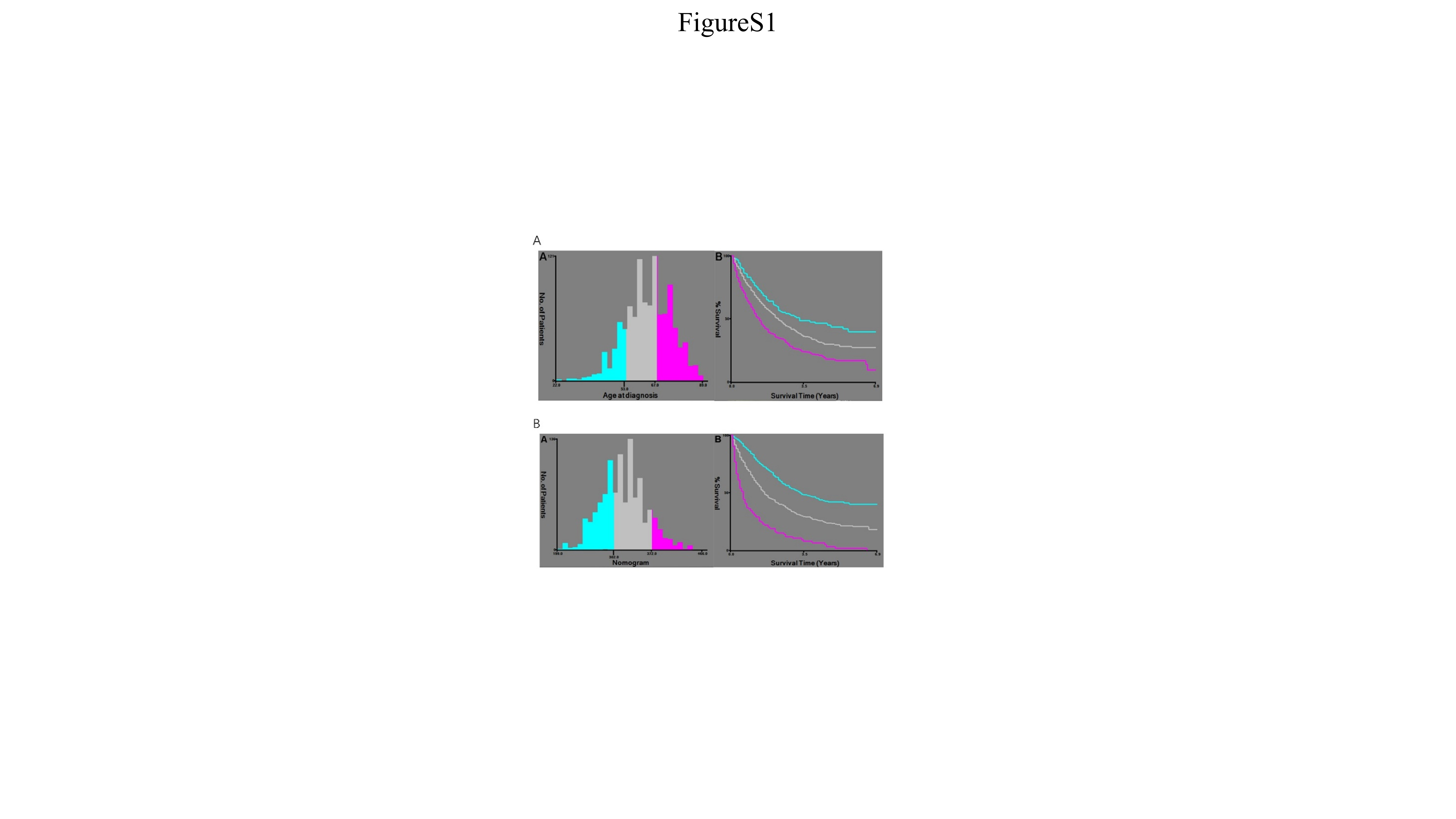

Supplement: Supplementary Figure 1 — X-tile program settings and results. [file Image_1.tif]

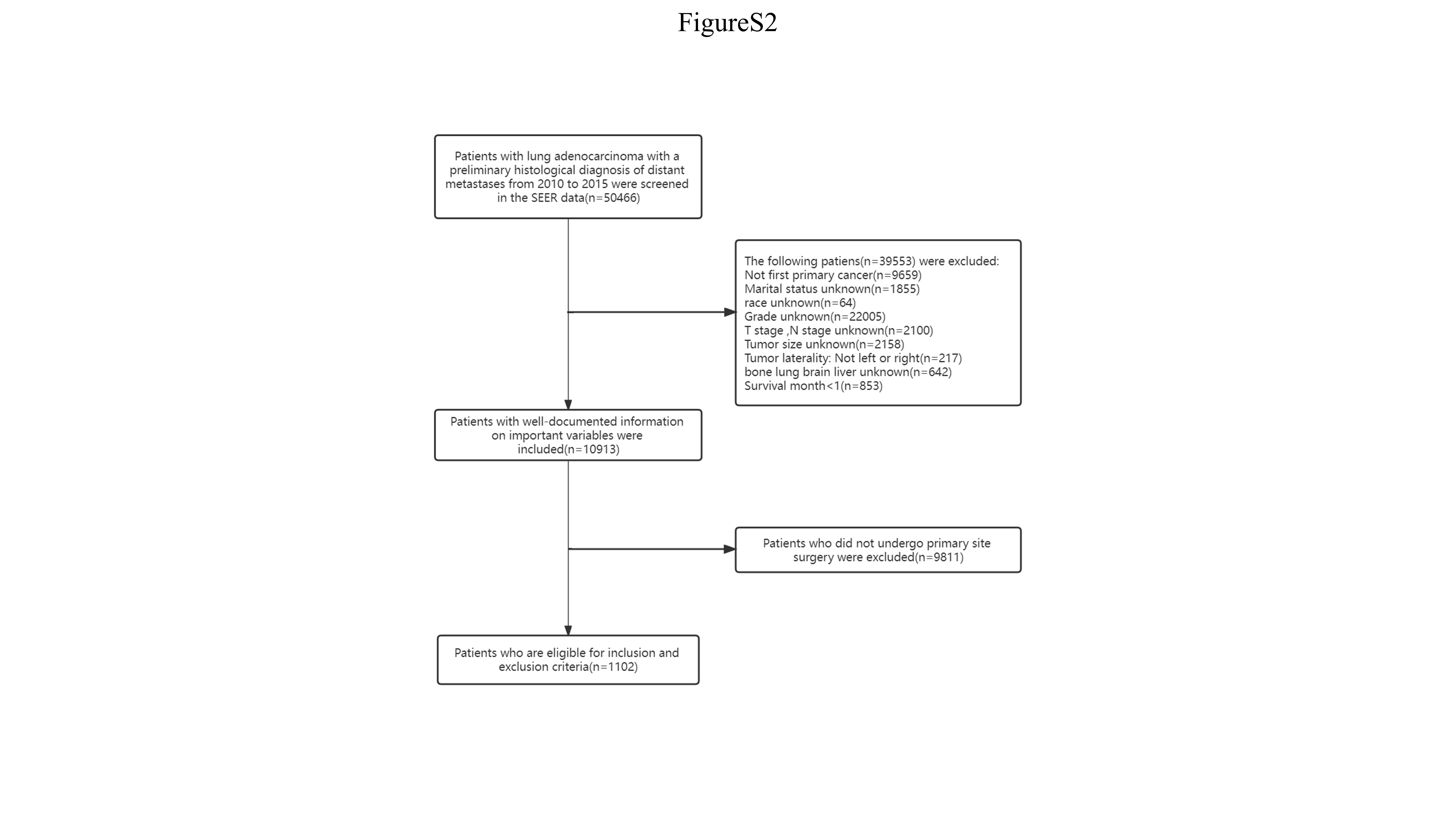

Supplement: Supplementary Figure 2 — The flowchart of patient selection. [file Image_2.tif]

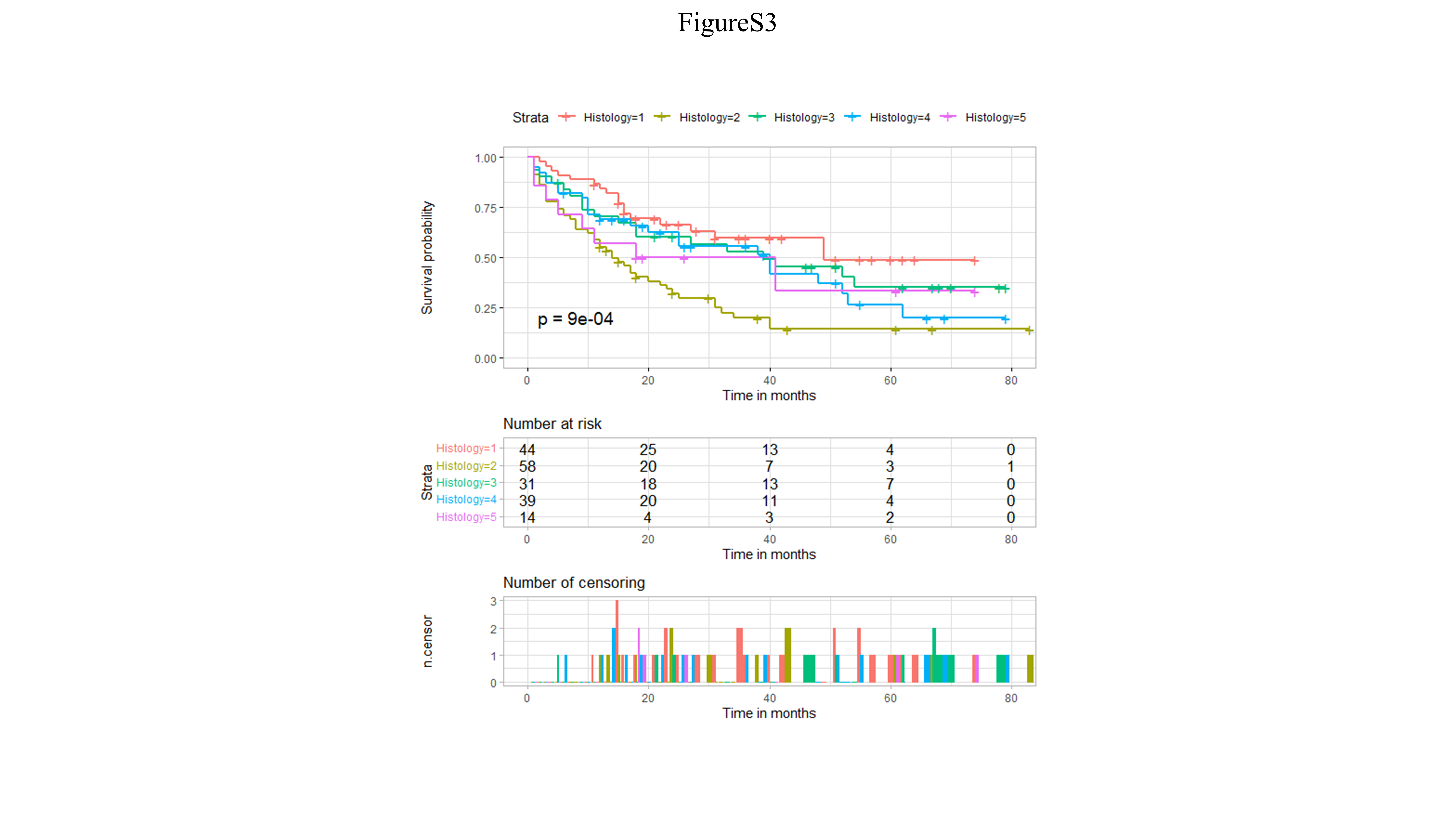

Supplement: Supplementary Figure 3 — Survival curve analysis of major histological subtypes and variants of invasive lung adenocarcinoma. Histology (1:Acinar-predominant;2:Colloid predominant;3:Lepidic-predominant;4:Papillary-Predominant;5:Solid predominant with mucin production) [file Image_3.tif]

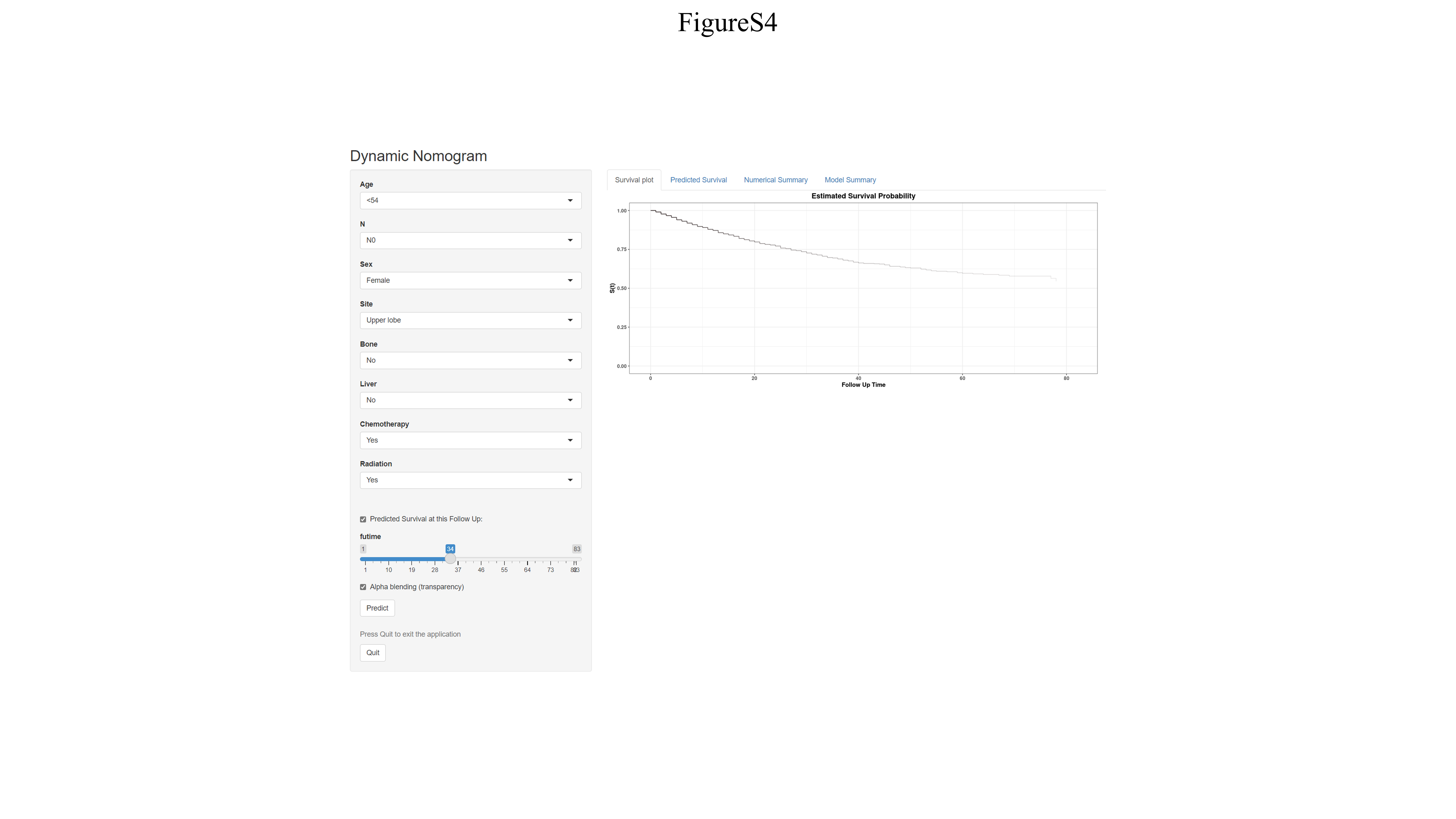

Supplement: Supplementary Figure 4 — Online web server interface for dynamic prognostic nomograms for patients with metastatic LUAD. [file Image_4.tif]
